# The Vicious Cycle: Problematic Family Relations, Substance Abuse, and Crime in Adolescence: A Narrative Review

**DOI:** 10.3389/fpsyg.2021.673954

**Published:** 2021-07-26

**Authors:** Valeria Saladino, Oriana Mosca, Filippo Petruccelli, Lilli Hoelzlhammer, Marco Lauriola, Valeria Verrastro, Cristina Cabras

**Affiliations:** ^1^Department of Human, Social and Health Sciences, University of Cassino and Southern Lazio, Cassino, Italy; ^2^Department of Education, Psychology, Philosophy, University of Cagliari, Cagliari, Italy; ^3^Department of Philology and Literature, LMU Munich, Ludwig-Maximilians-Universität, Munich, Germany; ^4^Department of Developmental and Social Psychology, University of Rome “Sapienza,” Rome, Italy; ^5^Department of Medical and Surgical Sciences, University of Catanzaro “Magna Graecia,” Catanzaro, Italy

**Keywords:** criminal behavior, family relationships, adolescence, substance abuse, risky behaviors

## Abstract

Despite the copiousness of studies on the risky behaviors of adolescents, we cannot establish with certainty the leading aspects involved in teens’ substance abuse and criminal actions. This review aims to explore the interplay among the family system, substance abuse, and criminal behavior. An analysis of the main results of the 61 articles published between 2010 and 2020 shows that adolescents whose parents are justice-involved and often absent from home are more likely to perceive lower cohesion, support, and poor family communication. These factors can involve them in criminal acts and substance abuse. Moreover, these conducts are often linked to a form of uneasiness and a search of autonomy. Indeed, risky behaviors could have more than one meaning. Our findings also suggest that the most diffused drug-related crimes in adolescence are economic crimes, weapon carrying, robberies, dealing, and drug possession. Considering these results, future clinical implications might be based on multidimensional approaches, focusing more on the family context to promote interventions for at-risk adolescents.

## Introduction

### Adolescence and Risky Behaviors

Adolescence is characterized by high psychosocial vulnerability (Hatano et al., 2018). It is a specific moment of the development of young people engaging in a great deal of personal and interpersonal exploration to understand themselves, their significant others, and their social world. Concomitantly, many physical, behavioral, and cognitive changes occur in the teenage years. Moreover, risk-taking behavior is common and is often associated with the engagement in unlawful acts and conducts (Gonzales et al., 2017).

Aggressive criminal behaviors in adolescence often aim to achieve autonomy (Piquero et al., 2013) and to build one’s identity, simultaneously modified by the family system and environment (Willoughby et al., 2014). The primary theoretical frameworks in criminology and developmental psychology suggest a multifactorial approach in the study of the topic. Indeed, risk-taking behavior in adolescence needs to consider individual, family, and environmental factors (Lösel and Farrington, 2012). According to these theories, risky behaviors can contribute to building adolescent’s self-image; therefore, if adolescents receive positive reinforcements about delinquency, antisocial behavior, or drug abuse, they are more likely to maintain these attitudes in adulthood (Jolliffe et al., 2017). These behaviors, in most cases, describe a form of uneasiness; in fact, an adolescent can communicate the feelings of rage, fear, and solitude and may show internalizing conducts, such as substance abuse, or externalizing conducts, such as illicit and aggressive actions against people or property (Moylan et al., 2010).

In this perspective, the family system assumes the role of protective or risk factors, especially referred to the family climate, communication, and parental support. According to this perspective, it is important to consider the meaning that risky behaviors could have for adolescents, especially in the study of delinquency and substance abuse conduct, which often involve young people (Johnston et al., 2017, 2018). This aspect is important to evaluate adolescents’ risky trajectories, incorporating the developmental perspective.

### Substance Abuse: Some Definitions

Juvenile involvement in risky behaviors continues to be a sensitive issue. Substance abuse is among the most common risky behaviors widespread in adolescence. For this reason, a definition of the term is needed.

Substance abuse could be described “as a maladaptive pattern of drug use leading to clinically significant impairment or distress” (Kpae, 2019). According to some authors (Mamman et al., 2014), the term usually refers to illegal drugs. However, substance abuse is also related to the degree of social acceptance of substances, such as alcohol, prescription medicine, and other legal substances, which are viewed as illicit and less harmful. Indeed, it may also be defined as the use of psychotherapeutic and medical drugs in the presence or in the absence of a specific diagnosis (Fareo, 2012). According to the Diagnostic and Statistical Manual of Mental Disorders DSM-III-R (American Psychiatric Association [APA], 1987), “psychoactive substance abuse” is defined as “a maladaptive pattern of use indicated by. continued use despite the knowledge of having a persistent or recurrent social, occupational, psychological, or physical problem that is caused or exacerbated by the use (or by) recurrent use in situations in which it is physically hazardous.” The definition of “substance abuse,” instead, is not included in the fifth version of DSM (American Psychiatric Association [APA], 2013). DSM 5 identifies the “Substance-related and addictive disorders” section, including “Substance use disorders,” “Substance-related disorders,” and “Substance/medication-induced mental disorders.” Moreover, due to the ambiguity in its definition, ICD-10 use the term “abuse” only in the case of non-dependence-producing substances. The WHO (WHO Expert Committee on Drug Dependence and World Health Organization, 1969) also highlights the same as the hazardous and harmful use of illicit drugs could result in the dependence syndrome that is derived from the repeated use of substances and leads to a “strong desire to take the drug, difficulty in controlling its use, (and) persisting in its use despite harmful consequences” (Kpae, 2019). In fact, both legal and illegal drugs have chemicals, which can influence the behavior and cognition of individuals.

There is consensus among researchers about the consistent and overdose intake of illicit drugs affects the brain and causes biological changes to the body. It creates the craving and excitement for things such as food and sex (Koob and Volkow, 2010) and can make, especially adolescents, not to weigh the consequences of their actions, affecting their development (Volkow et al., 2019).

### Theories of Drug Abuse

Mamman et al. (2014) proposed different theories explaining the origin of drug abuse: (1) sociocultural theory, (2) personality theory, (3) biological theory, and (4) learning theory. These theories describe the abuse of drug as influenced by several factors, emphasizing the personal motivation in using a specific substance. The key role of these theories is derived from the evaluation of the peculiarity of the relationship between the subject and the drug. This evaluation avoids any form of simplification and generalization in explaining drug abuse behaviors and might be useful for intervention and prevention programs based on drug abuse at different levels of influence.

The authors (Ibid) specified these theories in detail as follows:

(1) Sociocultural theory of drug abuse postulates that substance abuse is derived from the values shared by a specific society and context, which are culturally determined. For instance, in some cultures, adolescents are permitted to consume alcohol and smoke marijuana, and in other cultures they are not permitted to do so. Moreover, in such cultures, alcohol and tobacco are considered as a normal product, and used by youth and adults in everyday life and as a social activity or a way to spend time and have fun within the peer group, considering drug use as a life experience. Among some tribes in Nigeria, alcohol is also used in cultural activities (Mamman et al., 2014), whereas it is forbidden by the law in the north of Nigeria. Cultural norms and social attitude toward such drugs influence the personal and community perception of a specific substance. Foley et al. (2004), in a cross-sectional telephone survey among white, black and Latino youth, found that adults’ approval of alcohol use can increase or decrease adolescents’ drinking behavior according to the cultural and social perception of the context. As the social and cultural context promote the use of certain substances, the perception of risk decreases, especially among young people who tend to imitate the behavior of adults and to adapt to their context. This is also true for an illicit substance. Indeed, the data from an epidemiological study conducted in Australia indicates that the consumption of illicit substances by young people has recently increased as in United Kingdom. According to Howard Parker’s “normalization thesis” (Parker et al., 1998), youth no longer use drugs as a form of transgressive conduct but perceive this as a habit, which enriches their leisure time.” Today is no longer possible to attribute the use of drugs to a subcultural world described as bad or dysfunctional because drug abuse is more culturally normalized and ordinary. According to this theory, the inclusion of a concept introduced in the social cognitive theory by Bandura (1986) is needed. In fact, the etiology of the substance abuse can be described as the triadic reciprocity influence of the behavioral, environmental and personal factors.

(2) Personality theory of drug abuse considers specific personality characteristics more related to drug abuse. These personalities are characterized by elements, which could increase the possibility to use substances, such as low self-esteem, poor coping skills, low tolerance to frustration and in delay gratification, high sensitivity and impulsiveness, and a tendency of being emotionally dependent on others (Calamai, 2021). A few literature studies have shown that the onset of substance dependence tends to be earlier, especially in the presence of Cluster B Personality Disorder as reported by DSM 5 (American Psychiatric Association [APA], 2013). Antisocial, borderline, histrionic, and narcissistic personality disorder increase the likelihood of developing drug addiction (Lingiardi and Gazzillo, 2014) with a prevalence estimate of 75% for the borderline patients and of 95% for those with antisocial personality disorder (Hatzitaskos et al., 1999). Each personality disorder influences the motivations behind the use of a substance: each type of drug, in fact, produces different effects. According to a few literature studies, patients with narcissistic personality use cocaine as a “self-medication” to regulate dysphoric or depressed mood states, whereas people with antisocial personality and impulsivity traits, also defined as sensation seekers, use it to produce pleasant and positive emotional states (Rigliano and Bignamini, 2009). According to Khantzian (1997), cocaine and, in general, psychostimulants are used to regulate mood (mania, hypomania, and depression), whereas opiates are used to reduce the psychological suffering associated with negative emotions such as anger or sadness. Adolescents have some characteristics and traits common to the DSM cluster B personality disorders that increase the likelihood of consuming drugs, for example, impulsiveness, tendency to transgress, dependence in relationships, difficulty in delaying gratification, insecurity, difficulty in managing emotions and sudden changes in mood.

(3) Biological theory of drug abuse is based on the vulnerability link between potential genetic risk factors and the development of drug abuse. According to this theory, individuals diverge in taking drugs and in developing an addiction. For most people, drug use consists of single experience or a few experiences. Substance use among people who persist in taking drugs can be associated with specific situations, such as weekends or leisure time spend with friends, but remains an occasional behavior. People who use drugs more than occasionally can develop a drug addiction. In these subjects, the use of drugs is associated with a compulsive behavior directed to satisfy the physical and psychological need of the substance (O’Brien et al., 1986). Biological theory of drug abuse is divided from two points of view. The first one is a drug-centered approach, according to which drug addiction is derived from a repeated drug intake, which causes brain modifications and leads to tolerance, sensitization, and craving. Therefore, according to this point of view, the most vulnerable people are those who are most exposed to drug use due to a specific background (environments, neighborhoods, and peer groups). The second approach is individual-centered and describes substance abuse as a consequence of the pathological reaction to drug. Vulnerable people are those who have a specific biological substrate, which provokes this effect (Piazza et al., 1998). Specifically, research on the biological origins of individual vulnerability to addiction describes the key role of the mesencephalic dopaminergic neurons, stress, and glucocorticoids, as identified by Piazza et al. (1990). Also, all substances of abuse induce an effect on the neuronal system of the drug-reward circuit. The neurotransmission system that has been most clearly identified with the developmental actions of drug abuse is the mesolimbic dopamine system, with its efferent targets in the nucleus accumbens. The actions relevant to the reward of amphetamine and cocaine are in the dopaminergic synapses of the nucleus accumbens and possibly also in the medial prefrontal cortex. For instance, rats learn to press the lever for cocaine injections into the medial prefrontal cortex, which works by increasing dopamine turnover in the nucleus accumbens (Goeders and Smith, 1983, 1993). Understanding how our brain reacts to drugs is fundamental to establish the goals of addiction therapies and to better evaluate the physical addiction to drugs.

(4) Learning theory of drug abuse posits that drug abuse is derived from different types of learning (social, conditional, and instrumental). As mentioned in the sociocultural theory of drug abuse, Bandura (1986) explained the theory of social learning according to which people can learn by observing others. It is possible to apply this construct also to drug abuse (Niaura, 2000). Indeed, most people, especially youth, start in taking drugs due to the social conditioning or the peer pressure, learning this behavior through social interactions (family members, peer group, neighbor, and teacher). For instance, youth who frequently observe their parent relaxing in gambling or their peers who socialize and have fun after drinking alcohol, smoking marijuana, or after using other drugs could learn that these behaviors lead to positive results (Eiser, 2011). This perception is derived from a social learning. In addition, social interactions and the sense of acceptance are important factors for individuals who often see drug use as a way to develop or maintain social bonds. Moreover, people who only interact with addicted persons have fewer opportunities to learn healthy habits (Wilson, 1988). This aspect leads them to disengage from developing positive habitudes but progressively to occupy their time in using drugs. The learning associated with drug abuse influences the choice of social interactions. In fact, as the persons are encouraged to use drugs, they will look for like-minded people or groups and gradually move away from those who do not use drugs (Smith, 2021). This involves a psychological closure which risks becoming the future personality of the addicted subject. Learning theory of drug abuse could be useful in promoting the treatment based on a social learning perspective, with support groups and with an opportunity to interact with healthier people.

### Social Stigma and Poverty: Negative Identity and Crime

In adolescence, teens develop their identity, a coherent, homogeneous, and continuous image of the essence of their future personality (Erikson and Erikson, 2018). This process is derived from an interiorization and symbolization of models and the roles assumed during life experiences. A criminal or poor environment, offense involvement, justice-involved or drug abuser parents, relatives, or friends, and living in a poverty condition are some of the main social factors that can impact an adolescent development. Teens who suffered from one or more of these risk factors could receive negative expectations from others and interiorize a self-image based on the proposed perception of self. This is called “prophecy that is self-fulfilling” and derived from the labeling theory (McIntosh and Rock, 2018), which investigates the complexity and multidimensionality of individuals and their social interactions (Lemert, 1974). These concepts are linked to the stigma and the criminal stereotype (Ciampi, 2017).

According to this theory, juveniles labeled as “criminal” or “drug addicted” are more likely to suffer from marginalization, isolation, stigmatization, and imprisonment. They are confirmed of their negative identity by society, peer group, and family, creating a social stigma (Goffman, 1961; Chapman, 1968). In several cases, teens with justice-involved or drug abuser parents or adolescents who live in a disadvantaged neighborhood develop a sense of stigmatization, which leads them to assume criminal and antisocial conduct and to maintain it in adulthood (Adlaf et al., 2009; Luther, 2016; Massarwi and Khoury-Kassabri, 2017). Stigmatization is one of the most adverse consequences of parental incarceration (Phillips and Gates, 2011). Youth who have justice-involved parents are adversely affected and can develop emotional and behavioral issues (Al Gharaibeh, 2008). Similarly, drug abuse within the family negatively influences the perception of the adolescent on the substance and increment the others’ prejudice (Mehta and Farina, 2011). Goffman (1963) referred to this attitude with the term “stigma of courtesy,” which was also defined as secondary or associative stigma (Furst and Evans, 2015). Courtesy stigma indicates that the label affects the safety and well-being of family members, significant others, roommates, and others who are associated with a stigmatized individual, passing for association from the stigmatized people to the members of their social network (Goffman, 1963; Mehta and Farina, 2011).

Another theory explaining delinquency through the multifactorial interaction of factors is Wikstrom’ situational action theory (SAT) (Wikstrom, 2009). According to this model, it is important to evaluate the criminal actions by considering the individual, environmental, situational, and behavioral elements. Wikstrom analyzes an individual’s reactions to a given situation, asserting that the commission of a crime is derived from the interaction between the mentioned elements. Thus, criminality could be derived from desensitization, resulting in a natural tendency to act on the learned behavior (Ibid). Wikstrom conducted a longitudinal study on youth belonging to disadvantaged contexts, highlighting that the criminal context does not imply a tendency to commit a crime *per se* but rather stems from youth with prolonged exposure to it, thus, mostly influencing a deviant behavior (Wikstrom and Sampson, 2003). Similarly, Shaw and McKay (1942) developed the social disorganization theory to define the study of areas with a higher rate of crime and poverty as criminal areas. According to these studies, criminal areas consist of neighborhoods with a high need for economic assistance, unemployment, and general discomfort. Mostly, families decide to live in such conditions because they have no choice (Leventhal and Brooks-Gunn, 2000). These areas, therefore, become an attractive center for those who are looking for a permissive and an adequate environment for delinquent status (Krohn et al., 2009).

Despite these findings, we know that not all those who live in criminal and disadvantaged areas develop a deviant career, as shown by Oreopoulos’ study (Oreopoulos, 2003). The author studied two groups. In one group, the author analyzed the experiences of adults who were involved as children to a housing project in the Toronto metropolitan area. This area comprises a wide neighborhood location and services. The second group was composed of adults living in public houses with a higher exposure to crime and poverty. In comparing these two groups, Oreopoulos found that different living conditions did not play a significant role in determining the youth behavior and that the family factors were more powerful in influencing their behavior. Similarly, Jacob (2005) explored the experiences of youth relocated by the Chicago Housing Authority from public housing to private-market housing. The author found no evidence of the adverse effects of public housing poverty and disadvantage conditions, comparing the group of youths who moved in other places with the group of youths lodged in a public house. These results show the importance of considering other factors, such as individual and family elements, which may play a moderating role in the adulthood outcomes, especially during development.

Another factor influencing criminality is the socio-economic status (SES) (Hollingshead, 1975). In childhood and adolescence, low-SES neighborhoods could have an adverse influence on children’s and adolescents’ mental health and on externalized behaviors, such as acting out, aggression, drug use, and delinquent behaviors (Saladino et al., 2020b). According to the evaluation of the Yonkers Project, adolescents who remained in low-SES neighborhoods were more likely to become substance users, especially alcohol and marijuana users (Briggs, 1997).

Another evidence shows a strong connection between low-SES, drug use, and criminality in youth. Tobler et al. (2011) found a positive association between environment deprivation and the alcohol use. Tucker et al. (2013) examined the correlation between neighborhoods’ disorganization and the onset of drug use; based on the hypothesis that the onset for both alcohol and marijuana may be more likely among adolescents who come from a poor neighborhood, with greater residential instability and a poor perception of cohesion and safety. The first important result shows that residing in a neighborhood characterized by a high rate of unemployment is the most influential factor on adolescent’s onset marijuana use, whereas the environmental perception of disapproval in marijuana use is related to lower rates of adolescent cannabis use (Keyes et al., 2011).

Social stigma derived from justice-involved or drug abuser parent and a neighborhood with a high rate of crime and poverty could lead to delinquent behaviors to become a channel of transmission of criminal values, especially in childhood and early adolescence. Moreover, the criminal history of a neighborhood or of a family increases the social stigma and affects the probability of obtaining a higher education and employment, developing into the only possible career, criminality.

### Aim of This Study

This narrative review (NR) aims to: (a) document the reciprocal interaction among family relations, substance abuse, and criminal conduct and (b) evaluate the connection between substance abuse and criminality, focusing on the different types of offenses and drug-related conducts.

## Materials and Methods

To capture the complexity of the dynamic processes of family relations and adolescents’ behaviors, the following electronic databases were used in conducting this narrative review: Google Scholar, PsycINFO Database Record, and PubMed. Using the similar methods in previous reviews, we searched the databases using the following specific keywords to collect the studies dealing with the connection among family system, substance abuse, and criminal conduct: “family communication,” “drugs in adolescence,” “drug abuse,” “alcohol abuse,” “substance abuse,” “family and drugs,” “drugs and crime in adolescence,” “family influence,” “behavior and drugs,” “impulsive acts and family,” “family background,” “family support,” “parental support,” “family climate and substance abuse,” “juvenile delinquency,” “family climate and crime,” and “parent drug talk and substance abuse.” The search was conducted between March 2019 and December 2020. It is well known that narrative reviews do not include databases and inclusion criteria (e.g., Cipriani and Geddes, 2003; Collins and Fauser, 2005). However, to allow the readers to better evaluate the transparency of the work, we provided some key elements about the search strategy (Campo et al., 2019). The quality of a narrative review may be improved by borrowing from the systematic review methodological elements aimed at reducing bias in the selection of articles for a review and employing an effective bibliographic research method. Due to the existence of no consensus on the standard structure of an NR, we used the common format Introduction, Methods, Results, and Discussion (IMRAD) (Ferrari, 2015). Moreover, due to the absence of specific standard in the selection of the range of time for the research query and using similar methods of previous reviews (Weinberg et al., 1998; Putnam, 2003) the authors decided to focus their work on the past 10 years in writing the current review and also to avoid obsolete contributions.

For this reason, the current review includes the articles, reviews, and reports published in English between 2010 and 2020. The Articles that used the definitions of substance abuse as discussed in Section “Substance Abuse: Some Definitions” and the articles that conceptualized drug use as a risk factor in developing substance abuse conduct were included in our analysis, whereas the studies discussing drug misuse or drug addiction were excluded from the review.

Of the 150 articles returned from the search, 61 were retained for the current review after screening their titles and abstracts.

## Results

### Family Relationships, Substance Abuse, and Delinquency in Adolescence

Adolescents are more likely to be involved in behaviors such as drug abuse and criminal acts. These behaviors impact not only the individuals but also their family, schools, and social context. In adolescence, risky behaviors are common among individuals who have a problematic family system and difficulties in relationships with parents (Kam and Middleton, 2013), aggressive and risky acts among adolescents can be used as habitual ways to communicate. Such perpetual patterns of behavior need to be understood by going beyond the apparent meaning of risky actions (Saladino et al., 2020a).

Several studies emphasized the importance of family relationships and family climate—considering parental support and communication, parental drug abuse, and parental incarceration—on illicit and at-risk behaviors, such as crime and substance abuse of youth (World Health Organization, 2010; Piko and Balaìzs, 2012; Thomas et al., 2013; Buelga et al., 2017). Indeed, negative parent–children relationship could lead to a sense of lack support and to use violence as a strategy to survive (Kann et al., 2016). Adolescents who perceive their parents absent and unable to protect them from a risky context also experience a sense of un-confidence and discomfort (Garrido et al., 2018).

Along the same lines, the data from a brief review on the topic (Saladino et al., 2020b) confirmed these results, reporting that participants whose parents are involved in illegal activities and are often absent from home are more likely to experience a lack of family cohesion and support and are more at risk in developing binge drinking and smoking marijuana habits. On the contrary, the presence of parents increments the perceived support of the participants and, therefore, decreases risky conducts (Brown and Shillington, 2017). According to Tucker et al. (2013), in addition to the lack of closeness to parents, the availability of drugs at home and parents’ perception of alcohol and illegal drugs also are two variables within family risk factors, which can influence substance abuse conduct among youth.

The influence of these factors is associated with the family context change according to the substance. For instance, the abuse of marijuana seems to be more linked to the relationships with parents, as shown by a comparison between frequent marijuana users and never or less frequent lifetime marijuana users, where the first category of users is more likely to perceive a lack of parental support (De Looze et al., 2015). Binge drinking, instead, seems to be associated with less parental supervision and the absence of parent, the availability of alcohol at home, and parents who drink alcohol (Martínez-Montilla et al., 2020). The Canadian Institute of Health Research and the Heart and Stroke Foundation of Canada (De Looze et al., 2015) confirmed these results, showing an association between the frequency of alcohol consumption and parental drinking. Adolescents whose parents drink alcohol are more at risk in becoming alcohol and polysubstance users. This survey shows that the parent–children relationship assumes a mediator role in risky behaviors connected to substance abusive conduct among youth. Indeed, a positive relationship with parents might decrease the odd to develop an addiction in adulthood, and not depend on contextual variables, such as where adolescents attend schools in high- or low-at-risk neighborhoods (De Looze et al., 2015).

Regarding parent involvement in crime and its connection with an increased risk of substance abuse and delinquency, this factor is often related to a history of physical abuse, maltreatment, parental neglect, a negative family climate, which, as mentioned above, is strongly associated with criminality and substance abuse (Lee et al., 2012; LoBraico et al., 2020; Saladino et al., 2020b). The data from the 2016 Minnesota Student Survey on 126,868 youth in public schools found that parental incarceration could determine an increased occurrence of externalizing behaviors among teens (Ruhland et al., 2020). NeMoyer et al. (2020) confirmed these results, linking parental incarceration with self-reported delinquent acts of youth. Parental incarceration is also associated with an increased risk of developing substance abuse as the Baltimore Prevention Project discovered (Furr-Holden et al., 2011). According to this study, adolescents who have incarcerated fathers (13% of the sample) are more at risk of drug abuse and delinquency, compared with adolescents without justice-involved fathers (Furr-Holden et al., 2011). Kjellstrand et al. (2019) confirmed that parents formally incarcerated can lead to problematic developmental trajectories, such as suicide ideation, suicide attempt, adolescent delinquency, and substance abuse (Kjellstrand et al., 2019).

Good family communication and disclosure are also important in preventing risky behaviors among youth (Savage, 2014; Pettigrew et al., 2017). A strong sense of openness within the family context increases social and emotional skills among teens (Haverfield and Theiss, 2017), and decreases risky behavior (Harris et al., 2017; Massarwi and Khoury-Kassabri, 2017). Positive communication with parents is an antidrug socialization agent (Shin and Miller-Day, 2017). In this regard, parental drug-talk styles, especially in early adolescence, assume a key role in preventing substance abuse (Choi et al., 2017; Pettigrew et al., 2018). Shin et al. (2019) identified four types of parent’s drug-talk styles, considering Miller-Day and Kam (2010) parent–offspring drug-talk model (PODT): situated direct, ongoing direct, situated indirect, and ongoing indirect style. In the situated direct style of PODT, parents talk one time with explicit comments about substance use and abuse; in the ongoing direct style, parents express their opinion on drugs systematically; in the situated indirect style, they hint at the drug talk, showing their disappointment through non-verbal communication, and in the ongoing indirect style, they communicate repeatedly the opinions and feelings on drugs using verbal and non-verbal messages. Parents change their drug-talk style according to the specific age of their children and to the situation. According to the authors, parent drug-talk styles are influenced by family environments, which could be characterized by expressiveness, structural traditionalism, and conflict avoidance. The first dimension is characterized by openness and closeness, the second instead by a sense of control and power by parents and of obedience by children; and the third one tends to suppress conflicts to maintain family harmony. Shin et al. (2019) examined the relationships among parent–adolescent drug-talk styles, family communication environments, and substance use and abuse (alcohol, cigarette, marijuana, and chewing tobacco), and the main results of the study showed that the ongoing direct style has a more positive outcome than other drug-talk styles on substance abuse behaviors of youth. Regarding the family environment, compared to other dimensions, expressiveness can predict more positive family outcomes and interactions (Burns and Pearson, 2011), can influence more the personal anti-substance-use perception of youth (Shin and Miller-Day, 2017), and is significantly related to lower levels of use while conflict avoidance is related to higher levels of drug abuse.

Similarly, Reimuller et al. (2011) found that alcohol-specific communication characterized by permissive messages leads adolescents to a higher risk of alcohol abuse than alcohol-negative messages. Communication, in general, and parent drug-talk influence the personal perception of adolescents and their future choices in adulthood as well.

As suggested by the data, parental support, communication, and family climate can protect teens from risky behaviors (Haverfield and Theiss, 2017; Pereyra and Bean, 2017; Saladino et al., 2020b). It is important to consider the complexity of the relationship between parents and adolescents and their bidirectional influence on the developmental trajectories. An example of this perspective derived from the family-based therapy approaches that are focused on family in a multidimensional level. This approach is composed of three frameworks that help therapists and health professionals to think in terms of mutual interaction between different factors, including the influence on adolescents’ behavior. The first one is the protective factor framework, which includes family, social, and individual domains. Clinicians must know the interaction between these factors to facilitate positive adaptation during the main critical phases of the development. A framework of protective factors is based on interpreting each situation according to the current life circumstances of the adolescent’s family. The second one is a developmental perspective framework, which focuses on developmental psychology distinguishing the typical development from the dysfunctional development in terms of psychopathology and maladaptive behaviors. The last one is an ecological framework based on an ecological approach as it studies the human behavior in a specific context. The most used and effective family-based therapy considers and integrates these frameworks by taking care of the individual and his/her family system. One of the family-based models that have considerable effectiveness among teens involved in drug abuse and criminality is the multidimensional family therapy (MDFT). According to this model, the family is the principal arena and shapes the individual in both intrapersonal and intrafamilial dynamics through a modeling process, playing a role in reinforcing behaviors, both negative and positive (Liddle, 2010). For this reason, the family is considered as a starting point to improve the living quality of adolescents who present any form of behavioral problems. MDFT is based on indirectly helping family members to implement new ways of interacting with one another and supporting their family member’s drug-free lifestyle and facilitating a change of perspective. Furthermore, MDFT also involves other influencing systems, which maintain drug abuse, through the “extrafamilial” work. As reported by Liddle et al. (2011), MDFT engaged 97% of youth drug users as compared with 55% in services as usual (SAU) and retained 87% of these teens in at least 3 months after release compared with 23% in SAU. Compared to other approaches, MDFT has a higher long-term effect on both adolescent drug use and family functioning (Rowe, 2012). Another approach based on multilevel domains and focusing on the family system is the brief strategic family therapy (BSFT), which has an evidence base of effectiveness for drug abuse and related behavioral problems in adolescence. This approach, based on the work of Minuchin and Haley (Robbins et al., 2011), has a common perspective and the goals of MDFT, which aims at reducing adolescent behavioral problems by promoting good relationships among individual, family, and other systems, such as school and peers; supporting personal skills; and improving the new coping capacity in both adolescents and parents. Robbins et al. (2011) have conducted a study to compare BSFT with treatment as usual (TAU) including a sample of 480 adolescents and their family members using a specific and practical approach. The results showed that the group treated with BSFT reported higher levels of attendance and reduced probability to drop out than that treated with TAU. Moreover, participants treated with BSFT have shown a greater improvement in family functioning, also reported for adolescents, than that with TAU.

Finally, another model based on the behavioral and systemic approach is the functional family therapy (FFT). This model aims to identify and modify the maladaptive family patterns that maintain the problem. FFT changes the family interactions through positive reinforcements by introducing new problem-solving strategies. This therapy reduces recidivism among criminal teens and decreases antisocial behavior, as shown by a study on adolescent inmates with callous-unemotional (CU) traits who have committed violent and propriety crimes (White et al., 2013). The participants and their families were involved in FFT, and according to the evaluation pre–post treatment, the association between CU traits and recidivism was lower after the treatment as well as the aggressive and violent behaviors reported by parents (White et al., 2013). The same results were obtained by analyzing 917 families from both rural and urban settings in 14 different counties (Sexton and Turner, 2010). Most of the participants committed weapon crimes, had some gang involvement and a history of running away from home, were school dropouts, and used alcohol and marijuana. Criminal onset was in early adolescence between 12 and 17 years old. The participants were divided into two groups; one received FFT and the other TAU in traditional probation services in their local county. When the therapist was adherent to FFT, the results showed a reduction in serious crimes 1 year after treatment, and when the therapist was not adherent to FFT, the recidivism rates were significantly higher compared to the TAU group (Sexton and Turner, 2010).

The effectiveness of family-based models for adolescence drug abuse and other related problems has been demonstrated. The strength of these treatments is their focus on the interaction of several factors that trigger the vicious circle of crime and addiction in adolescence, and the results appear to have a broader outcome compared to the traditional family therapy approaches. Family-based therapy clients are less likely to be arrested, to relapse after a treatment, and to experiment internalizing and externalizing symptoms and in other co-occurring problems (Liddle et al., 2011). These data explained the reciprocal influence between parents and adolescents and highlighted the important implications for future clinical practice.

### Substance Abuse and Criminal Conduct

Drug abuse could influence criminal acts, including violent and non-violent offenses, such as threatening a person with a weapon, throwing objects, stealing money, physical aggression, sexual violence, and others (Coretta, 2012). Karofi (2012) confirmed the association between drug abuse and a high crime rate. Indeed, drug abuse could make individuals, especially adolescents, to commit crimes for acquiring drugs. Green et al. (2016) highlighted the link between substance abuse and economic crimes, weapon carrying, robberies (particularly among the users of heroin), illegal import, manufacturing, dealing, and drug possession.

Drug dealing is almost the most common crimes spread in adolescence. It could have different interpretations and motivations. The small drug dealing is often an extension of consumption and is understood as a sharing between friends who get the substance acquires prestige. Thus, the illegal act is specific to a certain phase of adolescence and does not continue into adulthood. Additionally, when drug dealing is associated with the youth culture and combined with personal and social problems, it may be used to have fun during a party with friends or to face feelings of sadness or insecurity. Adolescents who sell drugs in a specific context and situation are more likely to develop a drug addiction in adulthood rather than to become professional drug dealers. In other situations, the drug dealing may take place in non-disadvantaged social contexts, where the parents’ expectations lead the adolescent to look for alternative routes that become a sort of escape from family rules. Drug dealing can become dangerous for the development of an addiction rather than a criminal career because it is linked to a specific need for autonomy and recognition of one’s identity. On the contrary, when drug dealing is considered as a form of work and survival for those who come from contexts in which they do not perceive alternatives or when adolescents are hired by an organized crime group to carry out drug dealing activities, they are more likely to become professional drug dealers and to perpetuate this criminal behavior during adulthood (Johnston et al., 2018). Shook et al. (2013) documented that young drug dealers are also more likely to be engaged in other risky and delinquent behaviors. These results have been replicated in other studies with community and population samples (Vaughn et al., 2011a, b) and in research using the samples of justice-involved youth (Gunter et al., 2011; Magyar et al., 2011; Jeffrey et al., 2013).

The data suggest that the relationship between individuals and their context is fundamental for studying drug selling behaviors. Specifically, psychological, family, peer, and economic context are associated with differences among groups of young drug dealers. The literature studies converge in considering the drug-related conduct associated to antisocial behavior, delinquency, and criminality, including violent offending (Coretta, 2012). The choice of an individual to engage in such offenses, mostly exposing youth to incur in criminal sanctions, such as arrest. However, as the types and frequency of drug-related behaviors influence the link between drug-related behaviors and criminal offending, the explanatory mechanisms are complex to identify (Shook et al., 2013).

Additionally, to analyze the involvement of adolescents in drug-related behaviors, Phillips (2017) created a classification of these behaviors, considering the level of adolescents’ involvement measured on a continuum. Thus, it is possible to study the relationship between drug dealing and criminal conduct according to a categorical approach.

The link between drug abuse and criminal behavior was recently studied in an undergraduate student sample in Nigeria (Patrick and Okwukwe, 2019). Respondents of the survey affirmed that (the percentage of respondents is indicated in brackets): people who use drugs behaves aggressive (44.1%), use violent and bad language (9.5%), are school dropouts (9.2%), and are more likely to lose control (29.5%). Mostly, the respondents agree that substance abuse involves an organized crime (95%) and an armed robbery (97%), especially among youth, whereas 92% of the respondents think that substance abuse, among female students, leads to prostitution. Another study conducted in Nigeria (Mamman et al., 2014) confirmed these results, showing that adolescents who are drug abuser are more likely to be involved in gang formation and armed robbery.

The results of a few studies (Mamman et al., 2014; Patrick and Okwukwe, 2019) also showed some risk factors toward the development of drug abuse among students: peer group influence, curiosity, stress, family problem management, and high availability of the substance. Authors discussed the types and effects of this unhealthy practice among students, highlighting the need for the government, parents, teachers, counselors, and other members of the society to actively get involved in the fight against drug abuse not only by condemning it but also by living an exemplary lifestyle. The need for the proper education of parents on adolescent behavior was stressed out. The adolescent themselves should also be educated early enough about the dangers of addiction and parents on their side should try to watch their children very closely. By doing so, they can easily detect early issues in their own or other’s behaviors.

## Discussion

### Principal Findings

To summarize the key findings of this review, one needs to conclude that there has been an increased number of studies on the multifactorial interaction among the family system, substance abuse, and juvenile delinquency in adolescence. We also considered the associated factors of the family system such as family communication, feelings of disclosure, and family climate. The interaction among family, individual, and behavioral factors is not a linear but a circular process, which is defined by a reciprocal and an interactive influence. According to our findings, family relationships and climate (in particular, parental support and communication, parental drug abuse, and parental incarceration) have a strong impact on illicit and at-risk behaviors of youth, especially on delinquency and substance abuse (World Health Organization, 2010; Piko and Balaìzs, 2012; Thomas et al., 2013; Buelga et al., 2017). The family system represents the first space of communication and should give its members a feeling of belonging. When children live in disrupted or negligent families, they are more likely to be involved in risky conducts. However, these findings are not a norm but just the more common results. There may be cases, instead, where teens get involved in crime and drug abuse while not coming from dysfunctional families (Kann et al., 2016). In these cases, it is important to evaluate the individual and environmental factors that can influence, along with the family system, the adolescents’ behavior. Indeed, we focused on the interaction among influence factors according to a circular process, avoiding a linear and causal interpretation of behavior prediction (Lösel and Farrington, 2012).

Resorting to violence could be a strategy to survive at specific situations in which adolescents perceive parents as not supportive and absent in their lives (Lee et al., 2012; Kann et al., 2016; LoBraico et al., 2020; Saladino et al., 2020b). These negative feelings also contribute to developing poor confidence in others, negatively impacting the adolescents’ capacity to manage negative emotions (Garrido et al., 2018). Risky conducts become the only strategy to communicate their feelings and oversee their emotions (Myers et al., 2018). Another important finding is in regard to parent justice involvement, which is often associated with a lower perception of support and cohesion among family members, the absence of parents, and the lack of family communication. All these factors impact adolescent’s life, who might become a drug experimenter or emulate parents’ behaviors, being involved in criminal conducts (Saladino et al., 2020b). Furthermore, parents in prison negatively influence behaviors of youth, leading to more externalizing acts (Ruhland et al., 2020). This result shows a strong tendency of children to imitate parents’ behaviors, evaluating those conducts correct or justified. In this way, adolescents learn to react to frustration and to solve problems using violence and crime (Furr-Holden et al., 2011; Kjellstrand et al., 2019; NeMoyer et al., 2020).

Likewise, the availability of drugs at home and the perception of parents about drugs can influence substance abuse conduct among youth (Saladino et al., 2020a). Specially, binge drinking, more than marijuana use, seems to be related to low parental supervision and the absence of parent. This finding is interesting and may be related to the common use of alcohol among parents at home and with their low perception of risks in using it (Martínez-Montilla et al., 2020). Indeed, parental drinking is associated with more probability to become a drinker in adolescence and adulthood (De Looze et al., 2015). The use of drugs among youth is also related to parental drug-talk styles. Indeed, general good family communication and disclosure can prevent risky behaviors among youth (Savage, 2014; Pettigrew et al., 2017; Shin and Miller-Day, 2017), specifically, if parents are confident in talking about drugs (Choi et al., 2017; Pettigrew et al., 2018). According to the PODT (Miller-Day and Kam, 2010), the most favorable drug-talk style in preventing drug abuse is an ongoing direct style, in which parents express their opinion on drugs systematically (Shin et al., 2019). In the same way, a family environment characterized by expressiveness (openness and closeness among family members) could predict more positive family outcomes (Burns and Pearson, 2011), decreasing the risk of substance abuse and criminal acts more than other environments, such as structural traditionalism, and conflict avoidance, based on parents’ control and power, and a tendency to avoid conflicts (Shin and Miller-Day, 2017).

Regarding the connection between substance abuse and criminal conducts, our findings suggest that drug abuse and crime are associated, especially in the case of economic crimes, weapons usage, robberies, illegal import, manufacturing, dealing, and drug possession (Green et al., 2016). Drug dealing is a risk factor for the development of an addiction and for a criminal career and is often linked to a specific need for autonomy and recognition of one’s identity. Small traffics are transients and often an extension of consumption (Johnston et al., 2018). The evaluation of different contexts, situations, and family backgrounds is essential to give the correct interpretation of drug-related behaviors (e.g., escaping from family rules, parental negligence, survival, and involvement in peer groups). Families and schools are the primary prevention environments where the future of adolescents with drug abuse and criminal problems is shaped (Patrick and Okwukwe, 2019).

According to the main findings discussed above, drug abuse in adolescence is a complex problem, which involves the individual and social and environmental levels as revealed in developmental psychology and psychopathology research studies. Studies in this field have the common aim to identify the critical “markers” to prevent and treat the abuse of drugs as well as the illicit conduct diffused among both categories of teens: the convict and the student. Consistent research findings support the reciprocal interaction between positive family functioning and positive outcomes in desistance from crime and drug abuse in adolescents and adults (Hochstetler et al., 2010; Robertson et al., 2010).

### Limitations

This review presents some limitations. As noted, gender differences and social environment were not considered in criminal behavior and the abuse of substances. Moreover, even though the studies presented are European, the majority are conducted in the United States, probably because of the high rates of criminality and risky behaviors compared with other countries. Our review did not consider the influence of the culture in the main findings of research. Family relationships, the quality of communication, and support are also culturally determined.

This review did not consider recent changes in the illicit/licit drug definition, an important issue especially in the United States, in which most of the studies on crime and deviance are published. For example, recently, a change in the consideration of marijuana occurred: in reviewing a series of WHO recommendations on cannabis and its derivatives, the Commission on Narcotic Drugs (CND) removed cannabis from Schedule IV of the 1961 Single Convention on Narcotic Drugs—where it was listed alongside specific deadly, addictive opioids, including heroin, recognized as having little to no therapeutic purposes.

Another limit that emerged from our review is that different studies belong to the gray literature, therefore potential “spuriousness” problems could arise. For example, recently, the Marijuana Gateway Hypotheses were challenged (Jorgensen and Wells, 2021). MGH postulates that marijuana use serve as a “gateway” that increases the likelihood of engagement of users in subsequent use of harder and more harmful substances. Jorgensen and Wells (2021) make a call for improvements in the salience of findings regarding the MGH to better understand the complex relationship between marijuana and hard drug use and to discover if that relationship is causal. We consider this focus important not only for the MGH but also for deviance prevention: the circular approach adopted in this study allows to draw such connections. Jorgensen and Wells (2021) advanced the body of research on the MGH by applying propensity score matching (PSM) to a longitudinal, nationally representative sample. The authors appealed to the spuriousness problems applied to the whole body of research analysis: “In practice, control for all relevant confounding factors is an ideal that cannot be achieved, because the critic can always invoke the lack of control for important non-measured factors” (Kandel, 2003, p. 470). To solve this issue, they applied the PSM approach to provide an alternative to traditional approaches (e.g., regression analysis using unbalanced data) in testing the MGH when true experimental methods are not possible to be devised. PSM is a selection on observables method based on the counterfactual approach to hypothesis testing that allows researchers to estimate causal effects (Apel and Sweeten, 2010). This recent literature is very important to consider because our narrative review showed that one of the most used substances is marijuana.

We believe that future research linking family relations with drug abuse and deviance should be based on a solid experimental design to avoid spuriousness and to incorporate the recent advancements in drug classification.

Lastly, consistent with most narrative reviews, we did not appraise the quality of the included studies because our primary goal was to provide a broad perspective on the association of the family system, substance abuse, and crime in adolescence.

### Future Implications: A Multidimensional Approach

According to the main findings discussed above, drug abuse in adolescence is a complex issue, which involves individuals, the society, and families, as shown by research studies conducted in the developmental psychology and psychopathology field. As well as from the previous literature, our study has an aim at identifying the critical “markers” for juvenile risky and deviant behaviors, to prevent and treat the abuse of drugs as well as the diffusion of illicit conducts among teens. Consistent research findings support the reciprocal interaction between positive family climate and communication, and positive outcomes in desistance from crime and drug abuse in adolescents and adults (Hochstetler et al., 2010; Robertson et al., 2010; Okoiye and Adebisi, 2015). The family system plays a key role in the prevention of risky behaviors and social rehabilitation after being released from prison or after a drug addiction treatment.

These results could be useful in structuring programs on substance abuse and criminality prevention for the whole family system, both parents and youth. For instance, measures and programs focusing on adolescents are needed, based on the following indications and according to a perspective that considers the familial, educational, and social level and can be applied to both parents and youth:

(1) Familial interventions should be based on the direct involvement of parents, developing programs of support and parent training for families to improve their relationship with their children, and on decreasing the risk factors for youth associated with the dysfunctional family system. Familial interventions can help parents to establish positive communication, reduce conflicts within the family, promote a parenting style based on active listening, affection, and rules, lead parents to understand the effects of neglection, physical and psychological violence, and systematic exposure to a criminogenic context on the child’s development. These interventions could have a double aim: the treatment for families with drug-addicted and justice-involved parents or relatives and the prevention for conflictual families with dysfunctional communication and negative climate.

(2) Educational interventions for youth aim to develop more awareness among juveniles on the consequences of drug abuse and crime. These interventions could promote a joint and coordinated effort of family and school, which collaborate to reduce the spread of drug abuse and crime among youth. Educational projects can be the target considering gender and age differences. Teachers could organize lectures, seminars, rallies, and films, which teach alternative behaviors than the risky ones. Moreover, it could be useful to include a psychological support service at school for youth with family issues or who have parents involved in crime or drug addicted, to give a space in which they can disclose with a professional, reducing the risk to violent or self-harming acting out.

(3) Community interventions aim to promote social awareness through antidrug and anti-crime campaigns, especially on new drugs, drug selling websites on the dark web, legal consequences, and life in prisons. These interventions can use social media and social networks to diffuse a good policy on the topic and prevent from drug-addiction and juvenile justice involvement. It could be useful to establish a national and an international policy of intervention based on scientific divulgation of the main research findings to create best practices for schools and families in high-risk areas.

(4) Future researches are needed, especially focusing on longitudinal monitoring of the effect of long-term exposure to the risk factors mentioned in our and in the previous research. Also, psychologists, sociologists, and researchers in this field should be focused on identifying criminal areas in which young people are more likely to be exposed to violence, poverty, substances, and dissatisfaction with familial relationships to enrich the knowledge of the factors that can affect young people. Finally, it could be useful to stimulate a scientific debate with European and non-European countries to evaluate the topic according to a different cultural point of view and to enrich the discussion about possible interventions.

(5) Future studies are needed for addressing the cross-cultural dimension of the relationship of parents–children. Indeed, the development of theoretical models on parent–adolescent relationships reflect limited cultural considerations, in part due to the majority of previous works being carried out in western, educated, industrialized, rich, and democratic (WEIRD) countries (Dishion and McMahon, 1998; Stattin and Kerr, 2000). Nowadays, more and more research on parenting is conducted in different cultures as well as cross-culturally, calling for more nuanced tests of the degree of similarity/difference of parenting dimensions across cultures (Hadiwijaya et al., 2017). For example, recently, Vazsonyi et al. (2021), to address some research gaps, focused on maternal parenting excluding the role of father involvement, employed Steinberg and Silk’s (2002) conceptual framework to inform the operationalization of parenting, and tested its cross-cultural applicability by examining the links between both perceived maternal and paternal parenting processes and measures of adolescent internalizing and externalizing problems, which are risk factors for the development of deviant behaviors. This framework can be described by the interplay of three overarching dimensions, namely autonomy, harmony, and conflict. Three primary parenting domains, namely harmony, autonomy, and conflict, were mapped: harmony is operationalized as the affective dimension of parents–children relationship and includes constructs such as closeness, intimate communication, and warmth (Vazsonyi et al., 2021). Autonomy describes balancing growth and independence through connectedness and boundary-setting and includes monitoring, restrictiveness, and parents’ approval of the adolescent’s friends.

## Conclusion

The main findings of this review suggest that adolescents whose parents are involved in illegal behavior and the use of drugs are more likely to be involved in delinquency and substance abuse conduct. Furthermore, the perception of lacking family support, negative climate, and communication and of having justice-involved parents might contribute to the development of risky trajectories among young people. Moreover, there is a connection between criminal activities and substance abuse, which can lead to a future criminal career and addiction in later adolescence and adulthood.

The parent–children relationship seems to have a key role in risky behaviors among youth. Therefore, positive and supportive relationships with parents may reduce these risks. For this reason, we consider it useful for future research and interventions to place the focus on family in a multidimensional level. For instance, the so-called family-based approach therapy in treating and preventing drug abuse and criminal behavior among youth provides such possibilities.

Moreover, according to an overview of a systematic review about interventions for adolescent substance abuse (Das et al., 2016), school programs based on the promotion of social competence and the antidrug information were effective. However, there was poor empirical evidence in the evaluation of the long-term efficacy and the sustainability of substance abuse programs for adolescents.

Future research should focus on evaluating the effectiveness of specific intervention components with standardized intervention and outcome measures. Furthermore, the inclusion of gender differences in the family system influencing substance abuse and criminal conducts and of higher quality evidence, especially from low- and middle-income countries on effective interventions, to prevent and manage substance abuse among adolescents will be useful.

## Author Contributions

All the authors equally contributed to developing the project of present research, and to writing the manuscript. VS: conceptualization. VS, OM, and CC: methodology. VS and OM: investigation and writing—original draft preparation. LH, CC, ML, FP, and VV: writing—review and editing. VV, CC, ML, and FP: supervision. All authors have read and agreed to the published version of the manuscript.

## Conflict of Interest

The authors declare that the research was conducted in the absence of any commercial or financial relationships that could be construed as a potential conflict of interest.

## Publisher’s Note

All claims expressed in this article are solely those of the authors and do not necessarily represent those of their affiliated organizations, or those of the publisher, the editors and the reviewers. Any product that may be evaluated in this article, or claim that may be made by its manufacturer, is not guaranteed or endorsed by the publisher.
